# HBS1L deficiency causes retinal dystrophy in a child and in a mouse model associated with defective development of photoreceptor cells

**DOI:** 10.1242/dmm.050557

**Published:** 2024-07-30

**Authors:** Shiyu Luo, Bilal Alwattar, Qifei Li, Kiran Bora, Alexandra K. Blomfield, Jasmine Lin, Anne Fulton, Jing Chen, Pankaj B. Agrawal

**Affiliations:** ^1^ Division of Neonatology, Department of Pediatrics, University of Miami Miller School of Medicine and Holtz Children's Hospital, Jackson Health System, Miami, FL 33136, USA; ^2^ Division of Genetics and Genomics and The Manton Center for Orphan Disease Research, Boston Children's Hospital, Harvard Medical School, Boston, MA 02115, USA; ^3^ Department of Ophthalmology, Boston Children's Hospital, Harvard Medical School, Boston, MA 02115, USA

**Keywords:** Ribosomal rescue, HBS1L, Pelo, Retinal dystrophy, Photoreceptor cell

## Abstract

Inherited retinal diseases encompass a genetically diverse group of conditions caused by variants in genes critical to retinal function, including handful of ribosome-associated genes. This study focuses on the *HBS1L* gene, which encodes for the HBS1-like translational GTPase that is crucial for ribosomal rescue. We have reported a female child carrying biallelic *HBS1L* variants, manifesting with poor growth and neurodevelopmental delay. Here, we describe the ophthalmologic findings in the patient and in *Hbs1l*^tm1a/tm1a^ hypomorph mice and describe the associated microscopic and molecular perturbations. The patient has impaired visual function, showing dampened amplitudes of a- and b-waves in both rod- and cone-mediated responses. *Hbs1l*^tm1a/tm1a^ mice exhibited profound thinning of the entire retina, specifically of the outer photoreceptor layer, due to extensive photoreceptor cell apoptosis. Loss of Hbs1l resulted in comprehensive proteomic alterations by mass spectrometry analysis, with an increase in the levels of 169 proteins and a decrease in the levels of 480 proteins, including rhodopsin (Rho) and peripherin 2 (Prph2). Gene Ontology biological process and gene set enrichment analyses reveal that the downregulated proteins are primarily involved in phototransduction, cilium assembly and photoreceptor cell development. These findings underscore the importance of ribosomal rescue proteins in maintaining retinal health, particularly in photoreceptor cells.

## Teaser

**Summary:** Deficiency of the ribosomal rescue factor HBS1L causes retinal degeneration in both a human patient and hypomorph mice, and proteomic profiling reveals the underlying disruption of disc morphogenesis and photoreceptor cell function.

**Research Simplified**

Inherited retinal diseases are a group of conditions associated with vision loss caused by variants of genes involved in maintaining the function of the retina in the eye. In this study, the authors reported loss-of-function variants of the *HBS1L* gene that caused an inherited retinal disease in a young girl. The HBS1L protein is crucial for the production of other proteins in the cell and the authors used a Hbs1l-deleted mouse to investigate how it was causing impaired vision.

In mice with Hbs1l loss, the authors found that the retina was thinner than in normal mice due to loss of photoreceptor cells, which are responsible for the response of the eyes to light. The authors then performed a comprehensive analysis of all the proteins expressed in the retina of mice with Hbs1l deletion and found that the levels of 169 proteins were increased, whereas the levels of 480 proteins were decreased. An overview of the function of these proteins suggested that major biological processes in the retina were disturbed, such as the development of photoreceptor cells and the processing of signals from these cells to the brain in response to light. Therefore, the authors have uncovered the importance of HBS1L in fundamental retinal functions and how these functions go awry in inherited retinal disease.

## INTRODUCTION

Maintenance of cellular vitality necessitates continuous protein synthesis by ribosomes. However, various factors such as faulty mRNA production, inadequate availability of charged tRNAs and genetic errors can disrupt this process and result in detrimental cellular effects (Jamar et al., 2018; Brandman and Hegde, 2016). To mitigate these defects, organisms have evolved dedicated surveillance pathways designed to identify and prevent the accumulation of aberrant RNAs and proteins (Graille and Séraphin, 2012; Shoemaker and Green, 2012). One such mechanism is the ribosome-associated quality control (RQC) pathway, which encompasses two sequential steps, each addressing a distinct defect (Joazeiro, 2019). The initial step (termed rescue) involves sensing stalled ribosomes and facilitating the splitting of 80S subunits into 40S and 60S ribosomal subunits. The second step involves identification of 60S subunits obstructed with peptidyl-tRNA and the resolution of this aberrant structure. This leads to the release of free, translation-competent 60S ribosomal subunits and triggers the proteolysis of the nascent chain. During this process, efficient dissociation of stalled ribosomal subunits by rescue factors is crucial for optimal recruitment of downstream RQC components and nascent protein ubiquitination (Shao et al., 2013). The significance of this process in maintaining proteomic integrity and cellular fitness is underscored by the association of RQC machinery defects with various neurological diseases, such as Alzheimer's disease and Huntington's disease (Choe et al., 2016; Ishimura et al., 2014; Yang et al., 2016; Rimal et al., 2021).

Rescue factor Hbs1 [heat shock protein 70 (Hsp70) subfamily B suppressor 1], an ortholog of mammalian HBS1L in yeast, was originally identified for its ability to salvage stalled translation in yeast strains with reduced Hsp70 activity (Nelson et al., 1992). Human HBS1L, a paralogue of the canonical termination factor eRF3, is expressed ubiquitously and belongs to the GTP-binding elongation factor family (van den Elzen et al., 2014). Alternative splicing of *HBS1L* generates transcripts encoding two distinct proteins. The full-length HBS1L (referred to as HBS1L or Hbs1l henceforth) interacts with pelota (PELO or Pelo) to facilitate ribosomal release from stalled translation units (Shoemaker et al., 2010). In contrast, the shorter isoform encoded by the first four exons of full-length HBS1L and a unique last exon (exon 5a) positioned between exons 4 and 5 of the *HBS1L* locus interacts with the SKI complex and functions in global mRNA turnover (Kalisiak et al., 2017).

In previous studies, we identified a female child with biallelic pathogenic/likely pathogenic variants of *HBS1L* (hg19, chr6:135290431, NM_006620: c.1843C>T:p.R615X; chr6:135287466, NM_006620: c.2043+1G>T) leading to the loss of its longer isoform, resulting in a phenotype characterized by poor growth, microcephaly, facial dysmorphism, axial hypotonia, lax joints, neurodevelopmental delay, fused C2-C3 vertebrae, scoliosis, submucous cleft palate and retinal pigmentary deposits (Sankaran et al., 2013). Subsequently, we showed that *Hbs1l* hypomorph mice (*Hbs1l*^tm1a/tm1a^, with residual expression of wild-type *Hbs1l*) recapitulate several findings, including poor growth, facial dysmorphism and retinal pigmentary deposits (O'Connell et al., 2019). Additional studies in mouse models showed that *Hbs1l* is critical for mouse embryonic and cerebellar development but dispensable for granule cells after cerebellar development (Terrey et al., 2021). We have previously shown that depletion of Hbs1l leads to a tissue-specific reduction in its partner protein Pelo (O'Connell et al., 2019) – a finding subsequently described by others – which alters transcription regulation and reprograms the translatome (O'Connell et al., 2019; Terrey et al., 2021).

Inherited retinal diseases encompass a genetically diverse group of conditions in which variants in genes critical for retinal function are associated with progressive demise of photoreceptor cells and associated vision loss (Duncan et al., 2018). Currently, over 260 genes associated with inherited retinal diseases have been identified. Although most mutated genes are expressed specifically in the retina, a few ribosome-related genes (*RPL10*, *GTPBP1* and *GTPBP2*) have been identified that also cause neurological manifestations (Zanni et al., 2015; Ishimura et al., 2014; Terrey et al., 2020). In this study, we present the ophthalmologic findings observed in both the human patient carrying *HBS1L* recessive variants and *Hbs1l*^tm1a/tm1a^ mice, demonstrating that Hbs1l deficiency triggers extensive apoptosis of photoreceptor cells as early as postnatal day (P) 14 in mice and subsequent retinal degeneration. Comprehensive proteomic analysis in 4-week-old hypomorph mice illustrates that the loss of Hbs1l is associated with increase in the levels of 169 proteins (*P*≤0.05 and ≥1.5-fold change) and reduction in the levels of 480 proteins (*P*≤0.05 and ≤0.67-fold change). Gene Ontology (GO) biological process analysis and gene set enrichment analysis (GSEA) revealed that these reduced proteins are primarily enriched for ‘photoreceptor cell development’, ‘cilium assembly’, ‘phototransduction’ and ‘aerobic respiration’. Moreover, western blot analyses of *Hbs1l*^tm1a/tm1a^ mice at P14 demonstrated substantially decreased levels of select phototransduction and structural proteins, including rhodopsin (Rho) and peripherin 2 (Prph2), in addition to the Hbs1l-interacting partner Pelo. Taken together, these findings suggest a broad and early impact of the loss of Hbs1l and Pelo on key proteins for visual function and retinal health, in particular, proteins critical for rod photoreceptor cell development and function.

## RESULTS

### Retinal dystrophy in a child carrying *HBS1L* biallelic variants

We previously described a female child carrying compound heterozygous deleterious variants in the *HBS1L* gene, who presented with growth restriction throughout intrauterine and postnatal life, developmental delay and hypotonia (O'Connell et al., 2019; Sankaran et al., 2013). Because of concerns about limited visual responsiveness and tracking, serial ophthalmic examinations were conducted. At the age of 2 years, no eye abnormalities were found. By the age of 4 years, as previously reported (O'Connell et al., 2019), mild mottling of retinal pigmentation was noted. In her retina at the age of 7 years, multiple hypopigmented spots with soft margins were seen in a broad equatorial band (Fig. S1A). There were a few tiny, pigmented clumps among the hypopigmented spots. Optical coherence tomography (OCT) images (Fig. S1B) showed normal foveal architecture and, in extrafoveal retina, thinning of the outer nuclear layer (the optical correlate of photoreceptor nuclei) and an absent ellipsoid zone. Fundus appearance changed little over the ensuing years (Fig. S1C). She was 13 years old at her last examination, when corrected acuities were 20/50 for each eye and the dark-adapted visual threshold was elevated significantly – 1.9 log units above the normal mean (Hansen et al., 2017).

**Fig. 1.**
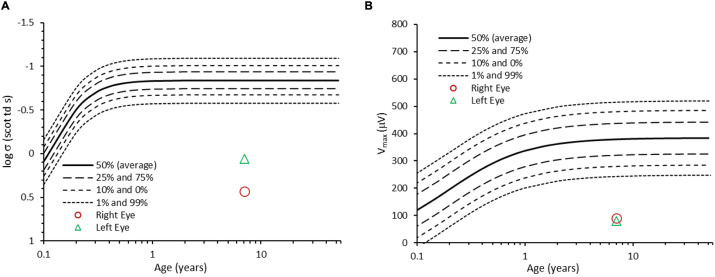
**Scotopic electroretinography response parameters.** The b-wave response parameters, log σ (A), an index of sensitivity, and V_max_ (B), the saturated amplitude, are shown for each eye of the patient. Also shown are the growth curves for normal development of log σ and V_max_ (Fulton and Hansen, 2000). For the patient, scotopic sensitivities (log σ) were +0.06 log scot Td s (left eye) and +0.43 log scot Td s (right eye), respectively; and saturated b-wave amplitudes (V_max_) were 80 µV (left eye) and 92 µV (right eye). Both log σ and V_max_ were significantly below normal for age. These parameters, derived from the electroretinography records shown in Fig. S2, were calculated using previously described analyses (Fulton and Hansen, 2000; Curran et al., 2023).

**Fig. 2.**
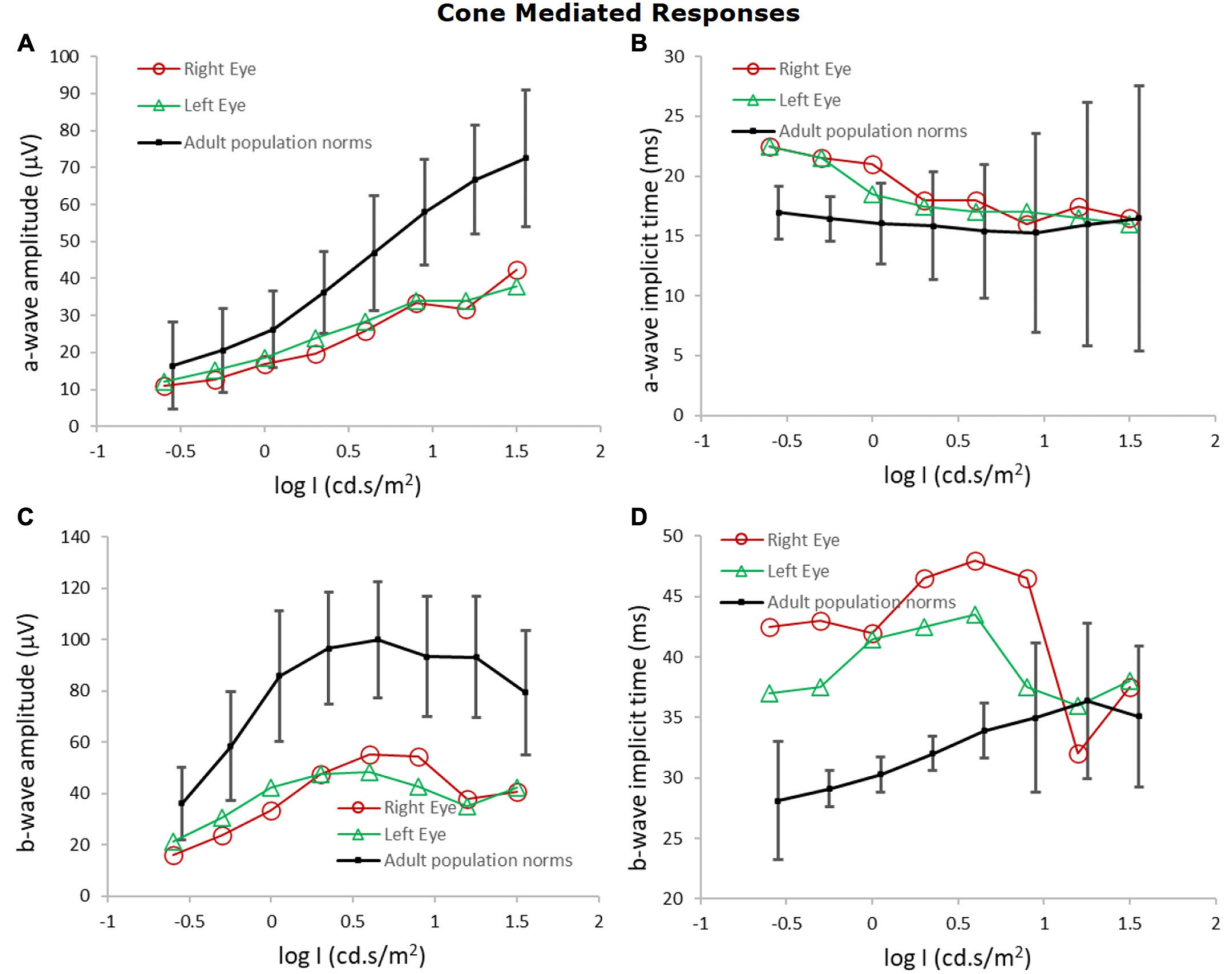
**Electroretinography shows impaired cone-mediated responses in the patient.** The amplitudes of the a-wave (A) and b-wave (B) responses were below the normal mean, and implicit times (C,D) were prolonged. With 30 Hz flickering white stimuli, the amplitude of the photopic responses were below the normal range (left eye: 46 µV, 37% of adult normal; right eye: 39 µV, 31% of adult normal). Data are expressed as mean±s.d. The normal mean is 125±40 µV and the lowest response amplitude in healthy controls is 52 µV. l, luminance.

At the age of 7 years, electroretinographic responses to full-field stimuli were recorded at an examination under anesthesia using previously described procedures and analyses (Curran et al., 2023; Hansen and Fulton, 2005; Fulton and Hansen, 2000). Electroretinography showed deficits in both dark- and light-adapted conditions that are indicative of generalized retinal dysfunction and retinal dystrophy. On records, such as those shown in Fig. S2, we measured the amplitude and implicit times of the a- and b-wave responses obtained under dark (scotopic) and light (photopic)-adapted conditions. The results are summarized in Figs 1 and 2. Scotopic b-wave sensitivity (log σ, where σ is the flash that evokes a half-maximum response) and saturated b-wave amplitude (V_max_) were both below the prediction interval of normal for age (Fig. 1). A-wave amplitudes were also attenuated and b-wave implicit times were prolonged. In photopic conditions, a- and b-wave amplitudes were below the normal mean and implicit times were prolonged; the amplitudes of the b-wave to the stimulus that ordinarily produces the maximum response (‘peak of the hill’) were less than 50% of the normal mean, as were the amplitudes of the responses to 30 Hz flickering stimuli (Fig. 2).

### Hbs1l is broadly expressed and enriched in photoreceptor cells, and its deficiency causes retinal degeneration in *Hbs1l*^tm1a/tm1a^ hypomorph mice

The RQC factors Hbs1l and Pelo are ubiquitously expressed in a variety of organs and tissues, such as the brain, spinal cord and skeletal muscles (O'Connell et al., 2019). To determine the transcript expression levels of *Hbs1l* in the retina, the web application eyeIntegration portal (https://eyeIntegration.nei.nih.gov) (Bryan et al., 2018; Swamy and McGaughey, 2019) was used, which incorporates single cell RNA-sequencing data from murine retinas across two studies (Macosko et al., 2015; Clark et al., 2019), enabling visualization of single-cell gene expression across multiple retinal cell types and different developmental time points [embryonic day (E) 11 to P14]. Our analysis of the data revealed that *Hbs1l* is broadly expressed in many types of cells during retinal development (Fig. S3). At P8, its transcript levels are relatively enriched in photoreceptor and bipolar cells, whereas at P14, its expression appears to be decreased and restricted to some of the photoreceptor cells. This suggests that Hbs1l is broadly expressed during retinal development and enriched in photoreceptor cells.

**Fig. 3.**
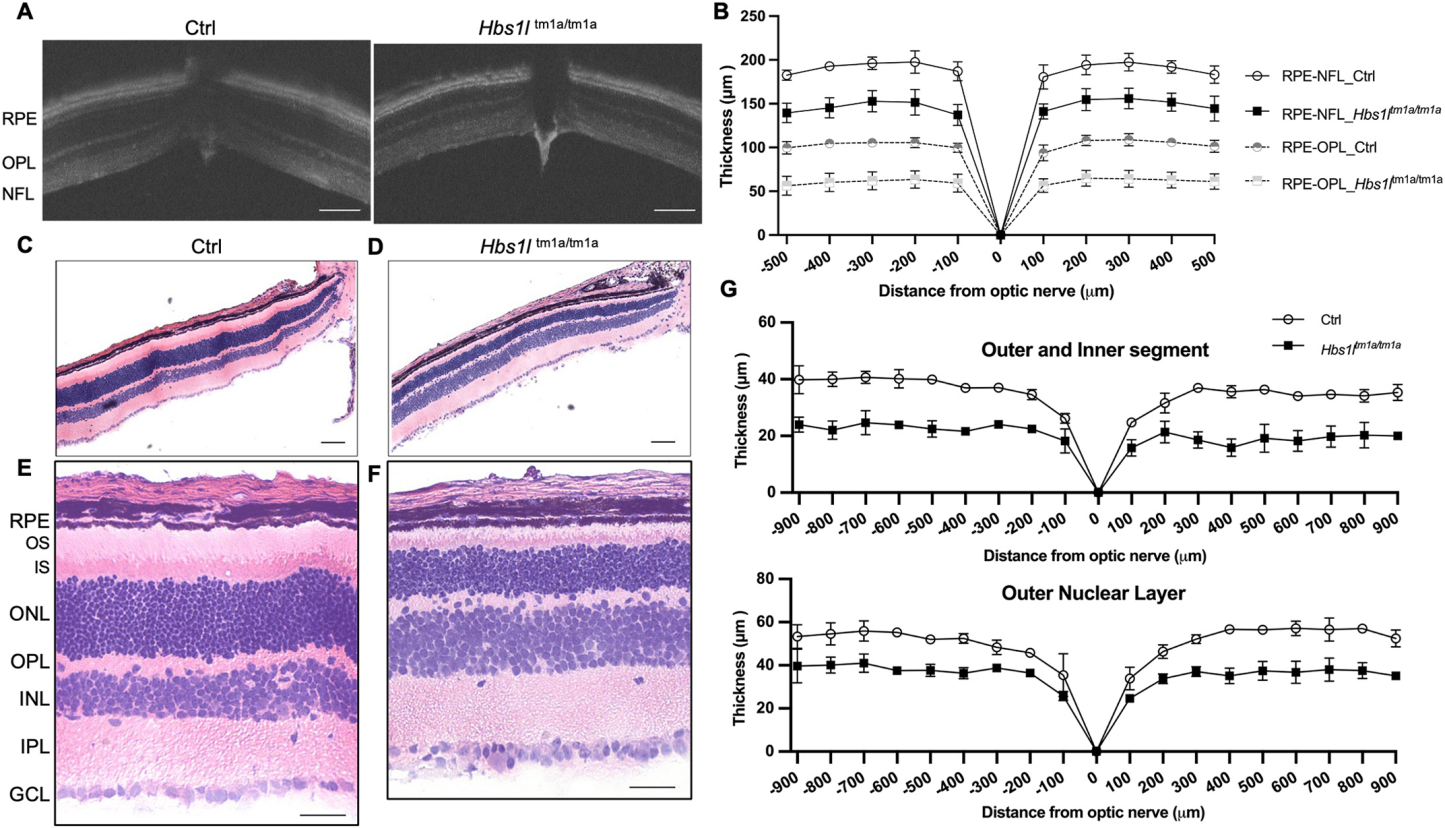
***Hbs1l*^tm1a/tm1a^ mice exhibited remarkable retinal dystrophy at 4 weeks of age.** (A) Representative retinal images from optical coherence tomography for thickness measurement. Scale bars: 100 µm. Ctrl, control; NFL, nerve fiber layer; OPL, outer plexiform layer; RPE, retinal pigment epithelium. (B) Quantification of thickness. Thickness of both the whole retina [distance from RPE to NFL (RPE-NFL)] and outer retina [distance from RPE to OPL (RPE-OPL)] was analyzed. Data are expressed as means±s.d. *n*=5 per group. **P<0.001 for all measurements (two-tailed unpaired *t*-test; not shown in the figure). (C-F) Representative retinal images of Hematoxylin and Eosin staining in control (C,E) and *Hbs1l* ^tm1a/tm1a^ (D,F) mice. Panels C and D are retinal segments between the optic nerve and approximately 1000 µm from the optic nerve. Panels E and F are retinal segments between 400 and 600 µm away from optic nerve. Scale bars: 100 µm. GCL, ganglion cell layer; INL, inner nuclear layer; IPL, inner plexiform layer; IS, inner segment; ONL, outer nuclear layer; OS, outer segment. (G) Measurements of thickness of the outer segment, inner segment and outer nuclear layer in control and *Hbs1l* ^tm1a/tm1a^ mice at 4 weeks of age. Data are shown as mean±s.d. *n*=3 per group. Significant differences were calculated using nonparametric Mann–Whitney test. ***P<0.0001.

A complete loss of Hbs1l leads to embryonic lethality (Terrey et al., 2021), but marked reduction of *Hbs1l* (*Hbs1l*^tm1a/tm1a^) is associated with a viable mouse model (Fig. S4A) which recapitulates several of the phenotypic findings present in our patient (O'Connell et al., 2019). As shown in Fig. S4B,D, expression of the *Hbs1l* full-length transcript (isoform I) and its protein levels were markedly reduced in the retina of this hypomorph mouse model. To evaluate the impact of Hbs1l loss on retinal structure and thickness, we performed OCT *in vivo* imaging in 4-week-old mice. The thickness of the whole retina, measured as the distance from the nerve fiber layer (NFL) to the retinal pigment epithelium (RPE) was decreased in *Hbs1l*^tm1a/tm1a^ eyes compared with that in the eyes of age-matched control mice (Fig. 3A,B; mean±s.d.: 134.0±44.42 µm versus 173.1±56.18 µm; *P*<0.001). The outer retinal layer, measured from the outer plexiform layer (OPL) to the RPE, was also thinner in *Hbs1l*^tm1a/tm1a^ eyes than in control eyes (mean±s.d.: 55.56±19.77 µm versus 93.92±30.72 µm; *P*<0.0001). Consistent with OCT imaging data, histological analysis at this age showed a significant reduction in the average thickness of the outer nuclear layer (ONL) or photoreceptor layer in *Hbs1l*^tm1a/tm1a^ mice (Fig. 3C-G; mean±s.d.: 34.15±9.28 µm versus 48.51±13.56 µm; *P*<0.0001). Additionally, the overall thickness of the outer segment (OS) and inner segment (IS) were also decreased in *Hbs1l*^tm1a/tm1a^ mice (Fig. 3G; mean±s.d.: 19.61±5.43 µm versus 33.62±9.22 µm; *P*<0.0001).

**Fig. 4.**
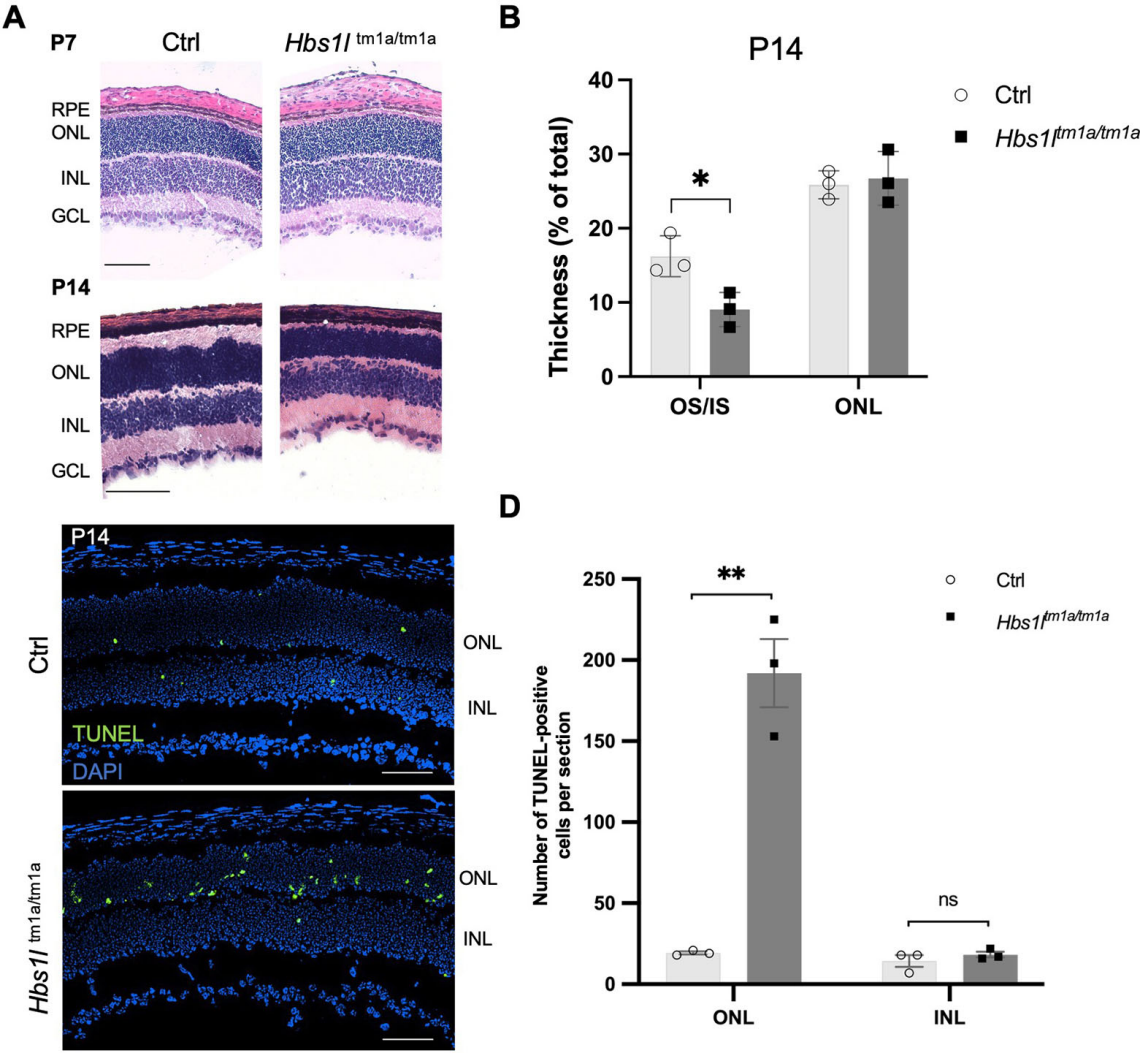
**Loss of Hbs1l leads to impaired development of photoreceptor cell and causes excessive apoptosis.** (A) Representative retinal images of Hematoxylin and Eosin staining in control (Ctrl) and *Hbs1l* ^tm1a/tm1a^ mice at postnatal day (P) 7 (top) and P14 (bottom). Scale bars: 100 µm. GCL, ganglion cell layer; INL, inner nuclear layer; ONL, outer nuclear layer; RPE, retinal pigment epithelium. (B) Quantification of the thickness of the outer segment (OS)/inner segment (IS) and ONL as a percentage of the total retinal thickness. (C) Representative images of retinal sections from 2-week-old control (*Hbs1l*^+/+^) and *Hbs1l*^tm1a/tm1a^ mice. TUNEL-positive cells are labeled in green; DAPI was used as a counterstain for cell nuclei. Scale bars: 100 µm. (D) Bar graph displaying the number of TUNEL-positive cells per section from 2-week-old control (*Hbs1l*^+/+^) and *Hbs1l*^tm1a/tm1a^ mice in the ONL and INL, respectively. Data represent mean±s.e.m. *n*=3 per group. Two-tailed unpaired Student's *t*-test was used for statistical analysis. **P<0.01.

To further explore the effects of Hbs1l deficiency on retinal development at earlier stages of development, we performed additional Hematoxylin and Eosin (H&E) staining in *Hbs1l*^tm1a/tm1a^ and control mice at P7 and P14. Although the whole retina of *Hbs1l* hypomorph mice appeared slightly thinner than that of control mice at P7, no obvious difference was noted in the thickness of the ONL or photoreceptor layer relative to that of the total retina (Fig. 4A; statistical analysis not performed due to limited number of pups). At P14, however, a thinner OS and IS layer was observed in *Hbs1l*^tm1a/tm1a^ mice, whereas the rest of the layers were similar to those in controls (Fig. 4A,B), suggesting impaired development of the photoreceptor OS and IS.

To test whether ONL thinning in the *Hbs1l*^tm1a/tm1a^ mice is associated with increased apoptosis of photoreceptor cells, we used terminal deoxynucleotidyl transferase (TdT)-mediated dUTP nick-end labeling (TUNEL) assay. At P14, the average number of apoptotic photoreceptor cells in the ONL of *Hbs1l*^tm1a/tm1a^ mice was significantly higher than that in the control group (Fig. 4C,D; mean±s.e.m.:105±87 versus 17±3, *P*=0.0012). In contrast, the number of apoptotic cells in the INL was comparable between *Hbs1l*^tm1a/tm1a^ and control mice. At 4 weeks of age, *Hbs1l*^tm1a/tm1a^ mice continued to show apoptosis in some photoreceptor cells in the ONL (Fig. S5), but it was much less than that at P14. Taken together, these findings suggest that loss of Hbs1l leads to retinal degeneration due to premature death of photoreceptor cells.

### Quantitative profile analysis of the retinal proteome in *Hbs1l*^tm1a/tm1a^ mice using multiplexed tandem mass tag

**Fig. 5.**
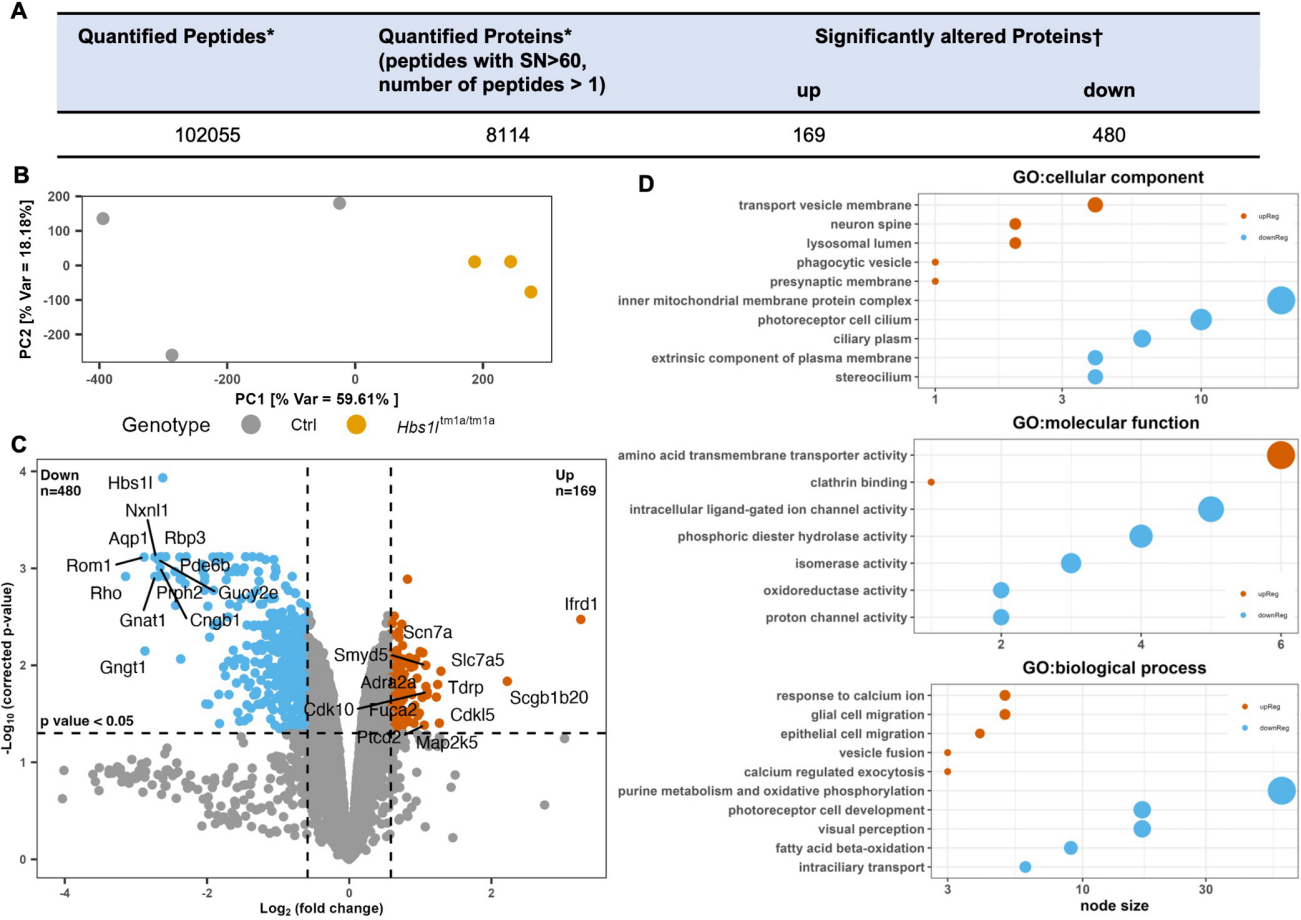
**Summary of retinal proteomics data and Gene Ontology analysis of significantly enriched proteins in *Hbs1l*^tm1a/tm1a^ compared to control mice.** (A) Table of quantified peptides and proteins in this experimental data set. Asterisks indicate quantification across all six channels with a signal to noise (SN) threshold >60 and number of peptides >1. The dagger indicates a Benjamini−Hochberg-corrected *P*-value of <0.05 and a fold change beyond ±1.5. (B) Principal component (PC) analysis of the biological replicates (*n*=3 per group). Var, variance. (C) Volcano plot displaying the −log_10_ (corrected *P*-value) versus log_2_ (fold change) for all quantified proteins. (D) A total of 649 DMPs (169 upregulated and 480 downregulated) were used for Gene Ontology (GO) analysis using g:Profiler. An enrichment map was created with FDR q-value<0.01, and combined coefficient>0.375 with combined constant=0.5. Clusters of nodes were labeled using the AutoAnnotate Cytoscape application. The top five GO clusters are shown for illustration; only two clusters were formed for GO: molecular function from upregulated proteins. The size of filled circles indicates the size of each cluster of nodes.

Loss of Hbs1l is known to alter transcriptional regulation and induce defects in translation elongation (Terrey et al., 2021), although its resultant proteomic changes have not been deciphered in retinas. In this study, quantitative mass spectrometry (MS) analysis of the retinal proteome in 4-week-old *Hbs1l*^tm1a/tm1a^ mice was performed and compared to that of littermate controls. A total of 8114 proteins from retinal tissue was quantified by multiple peptides at an initial protein false discovery rate (FDR) of less than 1% (Fig. 5A; see Table S1). The quality of the data and reproducibility of the biological replicates across groups were assessed using statistical metrics, hierarchical clustering and principal component analysis (Fig. 5B; Fig. S6).

To determine proteins that had significantly altered expression between *Hbs1l*^tm1a/tm1a^ and control mice, we used a *P*-value cut-off of ≤0.05 and at least 50% difference in levels and defined the proteins as differentially modulated proteins (DMPs, Table S2). This two-step differential analysis of *Hbs1l*^tm1a/tm1a^ versus control samples yielded 169 proteins with increased levels (*P*≤0.05 and ≥1.5-fold change) and 480 proteins with decreased levels (*P*≤0.05 and ≤0.67-fold change) (Fig. 5C). Following this, to examine the biological functions of DMPs, we performed enrichment analysis using GO and Reactome pathways (Table S3) using g:Profiler (https://biit.cs.ut.ee/gprofiler). The DMPs were significantly (*P*<0.01) enriched in the GO terms ‘inner mitochondrial membrane protein complex’, ‘photoreceptor cell cilium’ and ‘transport vesicle membrane’, and were involved in the molecular functions related to ‘oxidoreductase activity’, ‘proton channel activity’, ‘intracellular ligand-gated ion channel activity’ and ‘amino acid transmembrane transporter activity’ (Fig. 5D; Fig. S7). Furthermore, GO biological process analysis revealed that the DMPs in *Hbs1l*^tm1a/tm1a^ mice were mainly involved in processes related to ‘purine metabolism and oxidative phosphorylation’, ‘photoreceptor cell development’ and ‘visual perception’, which were also identified by Reactome pathway analysis (Fig. S8).

To explore the comprehensive effects of Hbs1l deficiency in the retina, we performed unbiased GSEA using the retinal proteomics data. Among 7775 gene sets categorized to GO biological process, 2715 gene sets were used in the analysis after gene set size filtering (minimum=15, maximum=250), of which 383 gene sets were altered with statistical significance (FDR q-value <0.05) and visualized with EnrichmentMap from Cytoscape (Fig. S9). The size of a node indicates the size of each gene set, and the edge indicates that two connected nodes share some genes. In accordance with GO and Reactome pathway analysis using g:Profiler, gene sets related to ‘aerobic respiration’, ‘photoreceptor differentiation’, ‘phototransduction’ and ‘cilium assembly’ were reduced, whereas gene sets related to ‘neurotransmission’, ‘synapse assembly’, ‘axonogenesis’ and ‘mononuclear cell migration’ were enriched in Hbs1l-deficient retina.

### Unique DMPs and validation using western blotting

Our proteomic data revealed that several proteins that participate in the phototransduction cascade of rod photoreceptors, such as rhodopsin, transducin subunits (Gngt1, Gnat1 and Gnb1), cGMP phosphodiesterase subunits (Pde6a and Pde6b), and cyclic nucleotide-gated channel subunits (Cngb1 and Cnga1), were among the most significantly reduced proteins (Fig. 5C; Table S2). Additionally, structural proteins essential for photoreceptor OS and disc morphogenesis, such as Prph2 and rod OS membrane protein 1 (Rom1), were also decreased. Interestingly, variants in rhodopsin, Pde6a, Pde6b, Prph2 and Rom1 are associated with inherited retinal degeneration in both humans and mice, and their downregulation may underlie the retinal degenerative phenotype observed in *Hbs1l*^tm1a/tm1a^ mice. Furthermore, apoptosis-associated proteins including Ddit3, Dapl1 and Dap were increased in the *Hbs1l*^tm1a/tm1a^ mouse retina.

**Fig. 6.**
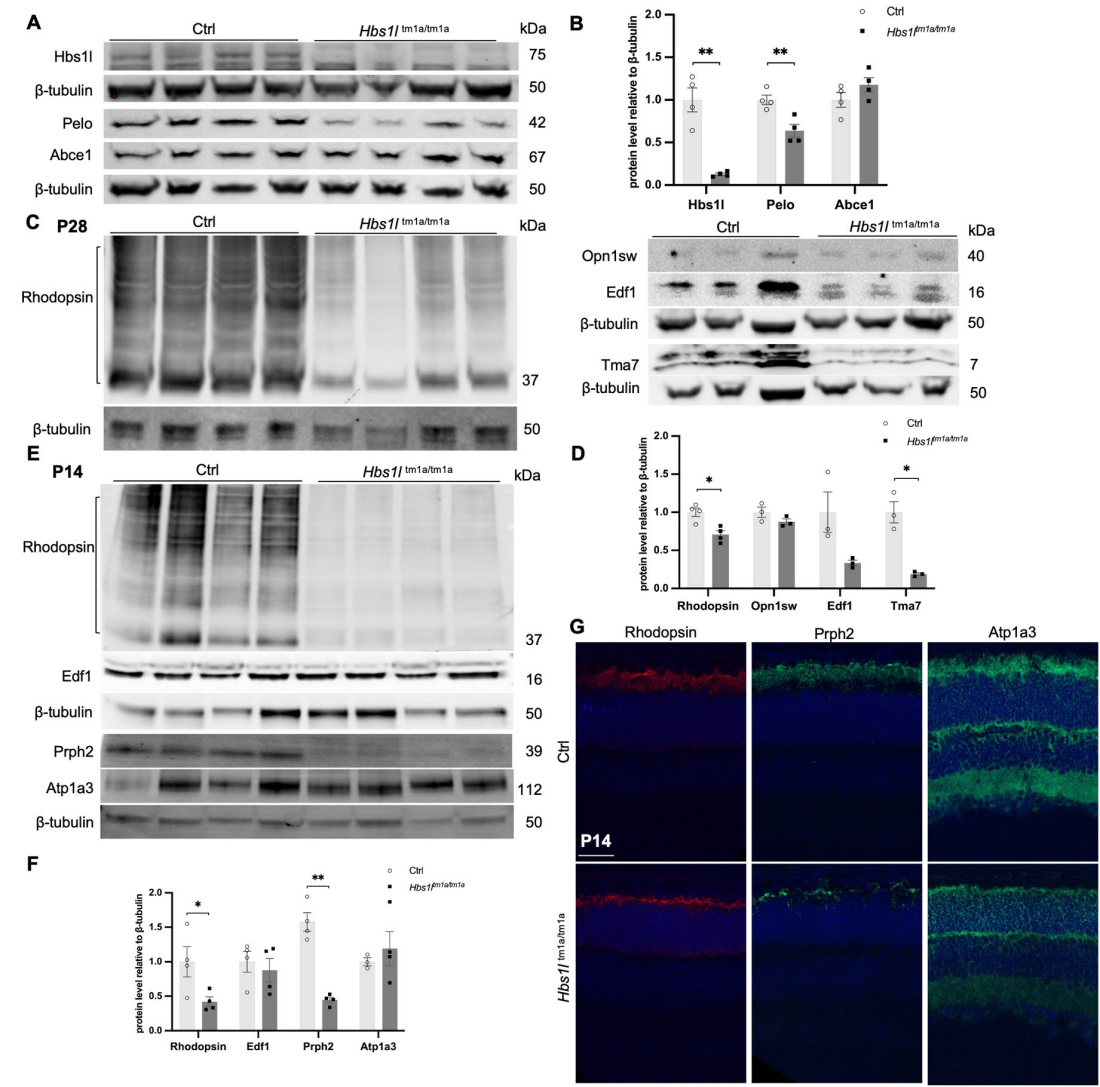
**Hbs1l deficiency leads to reduced protein levels of Pelo and the key rod proteins rhodopsin and Prph2.** (A-F) Representative western blot images (A,C,E) and quantification (B,D,F) by ImageJ analyses. (A,B) Hbs1l depletion was accompanied by decreased levels of its interacting partner Pelo, but the levels of the downstream ATPase Abce1 remained unchanged. (C,D) Western blot validation of select proteins identified to be downregulated from proteomic analysis in 4-week-old *Hbs1l*^tm1a/tm1a^ mice, including rhodopsin, opsin 1 short-wave sensitive (Opn1sw), endothelial differentiation-related factor 1 (Edf1) and translation machinery-associated protein 7 (Tma7). (E,F) Western blot analysis in 2-week-old *Hbs1l*^tm1a/tm1a^ mice revealed early reduction in the protein levels of rhodopsin and peripherin 2 (Prph2), whereas the levels of ATPase Na^+^/K^+^ transporting subunit α-3 (Atp1a3) and Edf1 appear similar between hypomorph and control mice. Data represent mean±s.e.m. *n=*3-4 per group. *P<0.05; **P<0.01 (two-tailed unpaired *t*-test). (G) Representative immunostaining images (*n*=1 mouse per group) of differential retinal proteins in 2-week-old *Hbs1l*^tm1a/tm1a^ mice. Scale bar: 100 µm.

On western blots, consistent with our previous findings from brain and many other tissues (O'Connell et al., 2019), loss of Hbs1l was accompanied by decreased levels of its interacting partner protein Pelo, whereas the level of the downstream ATPase Abce1 remained unchanged in mouse retinal tissue (Fig. 6A,B). Furthermore, in 4-week-old *Hbs1l*^tm1a/tm1a^ mice, western blotting revealed the remarkable decrease of rhodopsin levels but not of Opn1sw (Fig. 6C,D), a cone opsin involved in color vision, indicating that the observed retinal degeneration affects mostly rods but not cones at this time point. To distinguish whether these observed protein changes were directly driven by the loss of Hbs1l or derived from potential secondary effects due to photoreceptor cell degeneration, we further evaluated select proteins at an earlier time point, including rhodopsin, Prph2 and ATPase Na^+^/K^+^ transporting subunit α-3 (Atp1a3, enriched in IS of photoreceptor cells and neuronal cells). At 2 weeks of age, western blotting revealed significantly decreased rhodopsin and Prph2 proteins in *Hbs1l*^tm1a/tm1a^ mice, whereas Atp1a3 protein levels were similar to those of controls (Fig. 6E,F). Immunostaining confirmed a similar reduction of rhodopsin and Prph2 proteins, as well as a regional decrease of Atp1a3 protein in the photoreceptor outer layer (Fig. 6G). These findings suggest that Hbs1l deficiency directly compromises ribosomal synthesis of key rod photoreceptor proteins, resulting in impaired rod disc morphogenesis and/or OS renewal during photoreceptor cell differentiation and maturation, leading to retinal degeneration.

In addition to a list of proteins with retina-specific expression (Table S2), we also found decreased protein levels of translation machinery-associated protein 7 (Tma7) and endothelial differentiation-related factor 1 (Edf1). Tma7 is a ubiquitously expressed protein that may inhibit autophagy by activating the PI3K/mTOR pathway (Yang et al., 2023), and Edf1 is reported to coordinate cellular responses to ribosome collisions (Sinha et al., 2020; Juszkiewicz et al., 2020). Although western blots of 4-week-old Hbs1l-deficient retinas validated the reduction of both proteins (Fig. 6C,D), there was no difference in Edf1 levels between Hbs1l-deficient and control mice at 2 weeks of age (Fig. 6E,F). This indicates that the Edf1 level reduction is likely secondary and subsequent to the loss of photoreceptor cells.

## DISCUSSION

Historically, yeast Hbs1 and its mammalian counterpart HBS1L have been recognized as a ribosomal rescue factor and, through their interaction with PELO, play a critical role in processes of no-go decay (Doma and Parker, 2006; Shoemaker et al., 2010), non-stop decay (Tsuboi et al., 2012) and the dissociation of vacant or inactive ribosomes under stress (Pisareva et al., 2011; van den Elzen et al., 2014) to promote translation regulation. Additionally, they are also involved in nonstop protein clearance from organellar translocator channels for normal protein influx (Izawa et al., 2012), as well as in maintaining oxidative stress resilience (Jamar et al., 2017) and endoplasmic reticulum homeostasis (Guydosh et al., 2017). We have previously described a patient with *HBS1L* deficiency with a phenotype featuring growth restriction, facial dysmorphism and developmental delay (Sankaran et al., 2013; O'Connell et al., 2019). We have also shown that deletion of HBS1L is accompanied by decreased levels of the PELO protein, which contributes to an elevated baseline of 80S monosomes (O'Connell et al., 2019) and ribosomal pausing during transcription elongation (Terrey et al., 2021). Although disease modeling revealed a critical function of Hbs1l in mouse cerebellar development, little is known about the *in vivo* defects in other organs such as the visual system (Terrey et al., 2021).

In this study, we report that loss of HBS1L results in retinal degeneration in both a human patient and *Hbs1l*^tm1a/tm1a^ (hypomorph) mice. Bilateral fundus photography of the patient showed retinal pigmentation since she was 4 years old, and OCT at 6 years and 8 months of age showed a thinner ONL in extrafoveal retina and vanishing ellipsoid zone. Electroretinography at 7 years of age showed impaired scotopic and photopic responses. In 4-week-old *Hbs1l* hypomorph mice, OCT and H&E staining analyses indicate that the thicknesses of the total retina, ONL, OS and IS were decreased compared to those in controls. To evaluate the onset of this reduction in retinal thickness, we evaluated retinal structures at P7 and P14. At P7, a similar retinal structure was noted between *Hbs1l*^tm1a/tm1a^ and control mice, but by P14, the hypomorphs were noted to have a thinner photoreceptor OS and IS layer relative to the thickness of the total retina. This reduction was accompanied by increased apoptosis in the ONL as evident by the TUNEL assay. Consistent with the pathological phenotype observed in *Hbs1l*^tm1a/tm1a^ mice, proteomic analysis revealed a significant decrease in the levels of many critical retinal proteins, including photoreceptor visual signaling proteins (such as rhodopsin) and structural proteins (Prph2 and Rom1), along with elevated levels of apoptosis-related proteins.

The findings of OS and IS thinning at P14 suggest that OSs were probably not well formed, although increased apoptosis suggests degenerative process playing a role. We hypothesize that Hbs1l deficiency leads to impaired production of pan-retinal proteins, especially those required for proper disc morphogenesis such as Prph2 and Rom1. This blunted rod OS disc morphogenesis, as well as an imbalance between protein synthesis and degradation, sets the stage for photoreceptor cell apoptosis and retinal degeneration. In the meantime, without Hbs1l, the accumulation of dysfunctional ribosomes/proteins may overwhelm the lysosomes and autophagy pathways for protein degradation, thereby exaggerating the cell apoptosis.

One intriguing aspect is why decreased expression of ribosomal rescue proteins, such as Hbs1l, elicits tissue-specific phenotypes. Although *Hbs1l* hypomorph mice show retinal dystrophy, they do not display an overt neurodegenerative phenotype (Terrey et al., 2021). This may be due to the variable rates of protein synthesis and translation elongation among different organs, consistent with their metabolic rates (Gerashchenko et al., 2021). Thus, this retina-specific phenotype may be explained by the extraordinary demand of protein synthesis for disc membrane and OS renewal in the retina (Lewandowski et al., 2022; Chen et al., 2021; Barnes et al., 2021) combined with the relatively limited quantity of actively translating ribosomes due to Hbs1l depletion (O'Connell et al., 2019).

In summary, we report that genetic deficiency of *HBS1L* causes retinal degeneration in both a human patient and *Hbs1l*^tm1a/tm1a^ mice. We also present a comprehensive profile of the proteomic changes in the *Hbs1l*^tm1a/tm1a^ retina and reveal the underlying disruption of disc morphogenesis and photoreceptor cell function. These pathological consequences are likely caused by a failure of proper ribosomal recycling and attenuated protein translation following the loss of Hbs1l and Pelo, indicating future therapies to focus on restoring protein synthesis.

## MATERIALS AND METHODS

### Ethics statement

The Institutional Review Board at Boston Children's Hospital (Boston, MA, USA) approved the human subject study under the protocol 10-02-0253. Informed consent was obtained from all subjects or their legal guardians, and all clinical investigations were conducted according to the principles expressed in the Declaration of Helsinki. The Institutional Animal Care and Use Committee (IACUC) at Boston Children's Hospital approved our mouse work (approval number 16-06-3182R). The work followed the Guide for the Care and Use of Laboratory Animals and all of the regulatory protocols set forth by the Boston Children's Hospital Animal Resources at Children's Hospital (ARCH) facility.

### Patient evaluation

Widefield color fundus photographs were obtained using a RetCam Envision Ophthalmic imaging system (Middleton, WI, USA). More recently, widefield fundus images were obtained using the Optos imager (California; Optos, Dunfermline, Scotland, UK) including autofluorescence views. For spectral domain OCT (SD-OCT), we obtained horizontal B-scans (ART=100, 30° scan angle) through the macula using a Spectralis (Heidelberg Engineering, Heidelberg, Germany). ERG testing was performed after dark adaptation for 30 min using previously described procedures (Curran et al., 2023; Hansen and Fulton, 2005; Fulton and Hansen, 2000). In brief, we placed a bipolar Burian-Allen electrode (Hansen Laboratories, Coralville, IA, USA) on the eye and a ground electrode on the skin over the mastoid. Full-field stimuli were delivered using the Espion E2 system (Diagnosys, Lowell, MA, USA). Stimulus/response a- and b-wave records were obtained in both scotopic and photopic conditions from each eye and analyzed as previously described (Curran et al., 2023; Hansen and Fulton, 2005; Fulton and Hansen, 2000). From the records, we calculated log σ, the flash that evokes a half-maximum response, and V_max_, the saturated b-wave amplitude, and compared these to established normal values for age. The shape of the photopic a- and b-wave stimulus response functions were compared to established normal values for age. For ERG testing and fundus imaging, the pupils were dilated using combination drops: 1% cyclopentolate, 1% tropicamide and 2.5% phenylephrine (Leiters, Engelwood, CO, USA). ERG testing was performed and the RetCam images were obtained at an examination under anesthesia. The SD-OCT and Optos images were obtained in the clinic with the subject awake.

### Mouse strain and genotyping

The ‘knockout-first’ *Hbs1l*^tm1a^ [C57BL/6N-*A^tm1Brd^Hbs1l^tm1a(KOMP)Wtsi^*, MMRRC:048037] mice were obtained from the International Knockout Mouse Consortium [IKMC; Knockout Mouse Project (KOMP) Repository, IKMC project 79564; https://www.mousephenotype.org/data/alleles/MGI:1891704/tm1a(KOMP)Wtsi)], which is developed by the Wellcome Trust Sanger Institute (Hinxton, UK) (Dickinson et al., 2016). Mice were quarantined at Charles River Laboratories (Worcester, MA, USA) before import into our animal facility at Boston Children's Hospital. All experiments and quantifications were performed with at least three mice of each genotype and time point using mice of either sex. Absence of *Crb1* (*rd8*) mutation was confirmed by PCR to rule out potential confounding effects by *rd8* (Mattapallil et al., 2012).

Genotypes were confirmed by PCR using the *Hbs1l* forward primer (Hbs1l_F: 5′-TCTAATTCATGTGTGCCGCC-3′) and reverse primer (Hbs1l_R: 5′-TCCTGTGTTTTACCTGCATAGAGC-3′), which flanked the targeted exon 5, producing the wild-type PCR product of 483 bp. Addition of the targeting cassette-specific primer (Hbs1l_Cas: 5′-TCGTGGTATCGTTATGCGCC-3′) to the PCR master mixes to induce multiplex reactions generated a mutant-specific allele of 338 bp.

### Reverse transcription and quantitative PCR analysis

Adult mouse eye tissues were isolated and immediately frozen in liquid nitrogen. Total RNA was extracted with TRIzol reagent (Life Technologies). cDNA synthesis was performed on DNase-treated (DNA-free DNA Removal Kit, Life Technologies, AM1906) total RNA using oligo(dT) primers and SuperScript III First-Strand Synthesis System (Life Technologies). Quantitative real-time PCR (RT-PCR) was performed using iQ SYBR Green Supermix (Bio-Rad) and a CFX96 Real-Time PCR Detection System (Bio-Rad). Expression levels of β-actin (*Actb*) were used as input control for semi-quantitative RT-PCR. For quantitative RT-PCR analysis, expression levels of the genes of interest were normalized to β-actin expression levels using the 2^-ΔΔCT^ method (Livak and Schmittgen, 2001) and expressed as the fold change ±standard error of the mean (s.e.m.) relative to controls.

The primers for semi-quantitative RT-PCR were (F, forward; R, reverse): *Hbs1l* exon 3 F, 5′-GAAATTGACCAAGCTCGCCTGTA-3′; *Hbs1l* exon 6 R, 5′-CTCAGAAGTTAAGCCAGGCACT-3′; β-actin F, 5′-AGGCCAACCGTGAAAAGATG-3′; and β-actin R, 5′-AGAGCATAGCCCTCGTAGATGG-3′. The primers for quantitative RT-PCR were: *Hbs1l* F, 5′-AGACCATGGGATTTGAAGTGC-3′; *Hbs1l* R, 5′-CCGGTCTCAGGAATGTTAGGA-3′; *Hbs1l II* F, 5′-TGAAGTTGAACAAAGTGCCAAG-3′; *Hbs1l II* R, 5′-CTGCTTCCTCTGTGTTCCTC-3′; β-actin F, 5′-AGGCCAACCGTGAAAAGATG-3′; and β-actin R, 5′-AGAGCATAGCCCTCGTAGATGG-3′.

### OCT imaging

OCT imaging was performed as previously described (Wang et al., 2019; Gong et al., 2015). In brief, mice were anesthetized with a mixture of xylazine (6 mg/kg) and ketamine (100 mg/kg), and pupils were dilated with topical drops of Cyclomydril (Alcon Laboratories, Fort Worth, TX, USA). Two minutes after pupil dilation, lubricating eye drops (Alcon Laboratories) were applied to the cornea. Spectral domain OCT with guidance of bright-field live fundus image was performed using the image-guided OCT system (Micron IV, Phoenix Research Laboratories) according to the manufacturer's instruction and using the vendor's image acquisition software to generate bright-field images and OCT scans.

### Histology, immunofluorescence and TUNEL assay

Mouse eyeballs were enucleated, embedded in OCT Tissue Tek Compound (Sakura Finetek, USA), and placed in isopentane cooled by liquid nitrogen. H&E staining was carried out on 5 μm-thick cryosections obtained using a cryostat (Leica Biosystems, Nussloch, Germany). Immunofluorescence was performed using standard protocols with rabbit anti-Hbs1l polyclonal antibody (10359-1-AP, 1:50 dilution, Proteintech, Chicago, IL, USA), rabbit anti-Pelo polyclonal antibody (10582-1-AP, 1:100 dilution, Proteintech), mouse anti-rhodopsin antibody clone 4D2 (MABN15, 1:50 dilution, Millipore), rabbit anti-Prph2 polyclonal antibody (18109-1-AP, 1:50 dilution, Proteintech), rabbit anti-Atp1a3 polyclonal antibody (25727-1-AP, 1:50 dilution, Proteintech), and mouse anti-PKCα monoclonal antibody MC5 (MA1-157, 1:50 dilution, Invitrogen). TUNEL staining was performed using the TACS TdT *In Situ* Apoptosis Detection Kit (Trevigen, Gaithersburg, MD, USA) on retinal cryosections. Staining was carried out according to the manufacturer's instructions. The nuclei were counterstained with 4′,6-diamidino-2-phenylindole (DAPI). Images were captured using a Nikon Eclipse 90i microscope in conjunction with NIS-Elements AR software (Nikon Instruments, NY, USA). Six fields of view per section at 20× magnification was evaluated to determine the average number of apoptotic cells.

### MS using tandem mass tag

Retinal tissues from three pairs of *Hbs1l*^tm1a/tm1a^ mice and littermate controls at 4 weeks of age were isolated and MS was performed by the Thermo Fisher Scientific Center for Multiplexed Proteomics (TCMP) at Harvard Medical School, as described previously (Navarrete-Perea et al., 2018). Briefly, tissues were lysed by bead beating and proteins were quantified using the Pierce BCA Protein Assay Kit (Thermo Fisher Scientific). Samples were reduced with tris(2-carboxyethyl)phosphine (TCEP), alkylated with iodoacetamide and quenched with dithiothreitol. Proteins were precipitated using chloroform-methanol and reconstituted, and digestion was performed sequentially using LysC (1:50) and trypsin (1:100) based on the protease to protein ratio. Six-plex tandem mass tag-labeled peptides from six samples were mixed into one sample, desalted and fractionated with basic pH reverse-phase high-performance liquid chromatography, collected in a 96-well plate and combined to make a set of 12 fractions for liquid chromatography-tandem MS processing.

MS data were collected on an Orbitrap Eclipse Tribrid mass spectrometer (Thermo Fisher Scientific). MS/MS spectra were matched to a protein database using a SEQUEST-based in-house-built software platform (Navarrete-Perea et al., 2018). MS2 spectra were searched using the COMET algorithm (using a peptide mass tolerance of 50 ppm and fragment ion tolerance of 1.005 Da) against a mouse UniProt composite database (accessed 7 July 2022) containing its reversed complement and known contaminants. For whole-proteome analysis, only methionine oxidation was used as a differential modification, allowing up to two internal cleavage sites and up to three differential sites. Peptide spectral matches were filtered to a 1% FDR using the target-decoy strategy combined with linear discriminant analysis. The proteins were filtered to a <1% FDR and quantified only from peptides with a summed signal to noise threshold of >60.

To control for differential protein loading within a six-plex, the summed protein quantities were adjusted to be equal within each channel. Following this, relative protein abundance was calculated as the ratio of sample abundance to total abundance using the summed reporter ion intensities from peptides that could be uniquely mapped to a gene. Small differences in laboratory conditions and sample handling can result in systematic, sample-specific bias in the quantification of protein levels. To mitigate these effects, the relative abundances were log_2_-transformed and zero-centered at the protein level to obtain final relative abundance values, and then, we computed the median log_2_-transformed relative protein abundance for each sample and re-centered to achieve a common median.

### Bioinformatics and functional pathway analysis

Differentially expressed proteins were assessed using a two-sample two-tailed unpaired *t*-test (*P*≤0.05) and a fold-change threshold (≥1.5 for upregulation and ≤0.67 for downregulation). The reproducibility of all experiment output was evaluated by further statistical analysis such as overall data quality, unsupervised analyses such as clustering, and principal component analysis. All data analyses were performed using R (v4.2.1; https://www.r-project.org/) and RStudio (v2022.7.1.554; http://www.rstudio.com/) statistical software unless otherwise stated. Some specific libraries used for plotting include cowplot (v1.1.1; https://cran.r-project.org/web/packages/cowplot/), ggplot2 (v3.4.0; Wickham, 2016), ggrepel (v0.9.2; https://cran.r-project.org/web/packages/ggrepel/), gplots (v3.1.3; https://cran.r-project.org/web/packages/gplots/) and dendextend (v1.16.0; Galili, 2015). Network graphics were generated in Cytoscape (v3.9.1, National Institute of General Medical Sciences, Bethesda, MD, USA) (Shannon et al., 2003).

The GO of proteins was classified using g:Profiler (https://biit.cs.ut.ee/gprofiler) (Raudvere et al., 2019) to explore the functionality of altered GO biological process and reactome pathways in Hbs1l-deficient retinas. A cutoff of *P*<0.05 was adjusted for all GO categories. GSEA was performed using GSEA software (Subramanian et al., 2005; Mootha et al., 2003) and the Molecular Signatures database (MSigDB) (Liberzon et al., 2015; Liberzon et al., 2011). GSEA results were reduced and summarized by using the EnrichmentMap Pipeline Collection (v1.1.0) from Cytoscape (Morris et al., 2011; Oesper et al., 2011; Merico et al., 2010; Reimand et al., 2019).

### Western blotting

Retinal tissues from *Hbs1l*^tm1a/tm1a^ mice and littermate controls were dissected, snap frozen in isopentane and stored at −80°C until analysis. Protein isolation and western blot procedures were performed as described previously (Li et al., 2022). Proteins were probed with antibodies against Hbs1l (10359-1-AP, Proteintech, 1:500 dilution), Pelo (10582-1-AP, Proteintech, 1:500 dilution), Abce1 (ab32270, Abcam, 1:1000 dilution), rhodopsin (MABN15, Millipore, 1:500 dilution), Opn1sw (sc-14365, Santa Cruz Biotechnology, 1:500 dilution), Tma7 (20393-1-AP, Proteintech, 1:500 dilution), Edf1 (12419-1-AP, Proteintech, 1:500 dilution), Prph2 (18109-1-AP, Proteintech, 1:500 dilution) and Atp1a3 (25727-1-AP, Proteintech, 1:500 dilution). Quantification of protein levels normalized to β-tubulin levels (12004165, Bio-Rad, 1:4000 dilution) was performed using ImageJ software.

### Statistical analysis

Data were analyzed with GraphPad Prism (v.9.0; GraphPad Software) and reported as mean±s.d. or mean±s.e.m. Unpaired two-tailed Student's *t*-test was used for statistical analysis unless indicated otherwise. Fig. 4 used nonparametric Mann–Whitney test for group comparison of retinal layer thickness. *P*<0.05 was considered significant. The numbers of samples per group (*n*) and statistical significance for all comparisons are specified in the figure legends.

## Supplementary Material

10.1242/dmm.050557_sup1 Supplementary information

Table S1. List of 8114 identified protein groups in retina samples of control and *Hbs1l*^tm1a/tm1a^ mice with less than FDR 1% of proteins.

Table S2. List of 649 differentially modulated proteins in *Hbs1l*^tm1a/tm1a^ mice compared to controls. Proteins that met the p-value cut-off (≤0.05) and at least 50% difference were considered as differentially modulated proteins (DMPs).

Table S3. Gene ontology and pathway enrichment analysis of DMPs in *Hbs1l*-deficient retina using g:Profiler. (A) Cellular component, (B) Molecular function, (C) Biological process, (D) Reactome pathway are listed.
