# Supplementary material for: HBS1L deficiency causes retinal dystrophy in a child and in a mouse model associated with defective development of photoreceptor cells
Source: Dis Model Mech. 2024 Jul 30;17(8):dmm050557. doi: 10.1242/dmm.050557 (PMC11317091; doi:10.1242/dmm.050557)
Supplement: Supplementary information [file dmm-17-050557-s1.pdf]

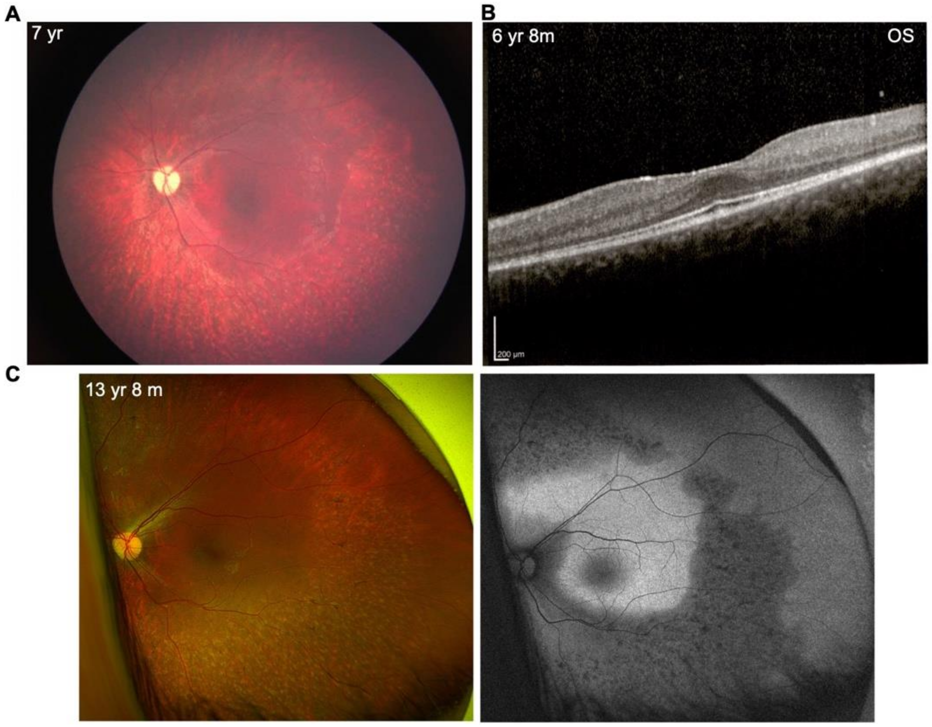

Fig. S1. Fundus and optical coherence tomography (OCT) images of the patient at different age. (A) Wide field photograph taken using RetCam at the patient's 7 years of age. **(B)** OCT image shows preserved foveal architecture and in extrafoveal retina a thin outer nuclear layer (the optical correlate of the photoreceptor nuclei) and absent ellipsoid zone. OS: left eye. Right eye image was similar (not shown). **(C)** Widefield images, including autofluorescent (right panel) obtained at her last visit using Optos imager. Right eye similar.

Disease Models & Mechanisms • DMM • Accepted manuscript

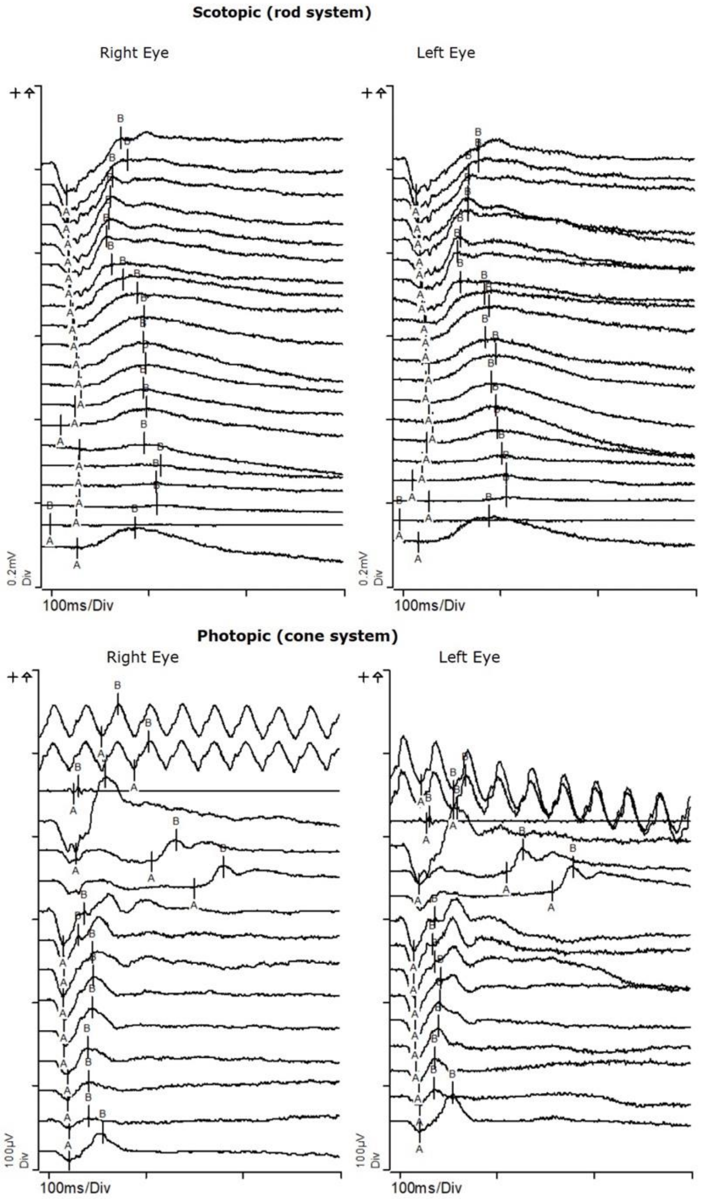

Fig. S2. Raw ERG traces of scotopic (upper panel) and photopic (bottom panel) response data from both eyes.

Disease Models & Mechanisms • DMM • Accepted manuscript

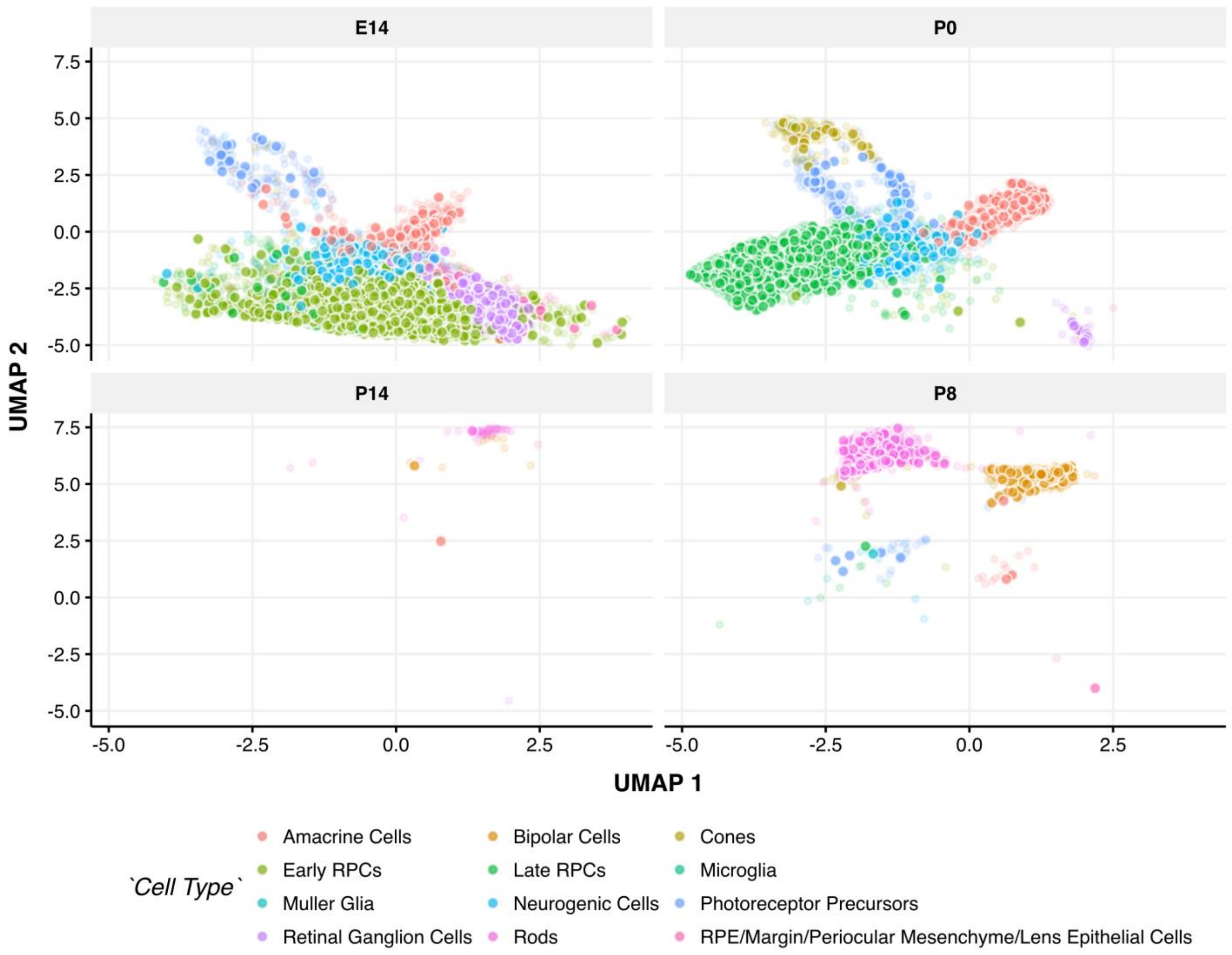

Fig. S3. Visualization of single cell mouse retina expression patterning (UMAP based) of *Hbs1l* gene. Query of *Hbs1l* transcript expression from the Clark et al. mouse retina single cell RNA-seq dataset on the web application portal eyeIntegration (https://eyeIntegration.nei.nih.gov).

Disease Models & Mechanisms • DMM • Accepted manuscript

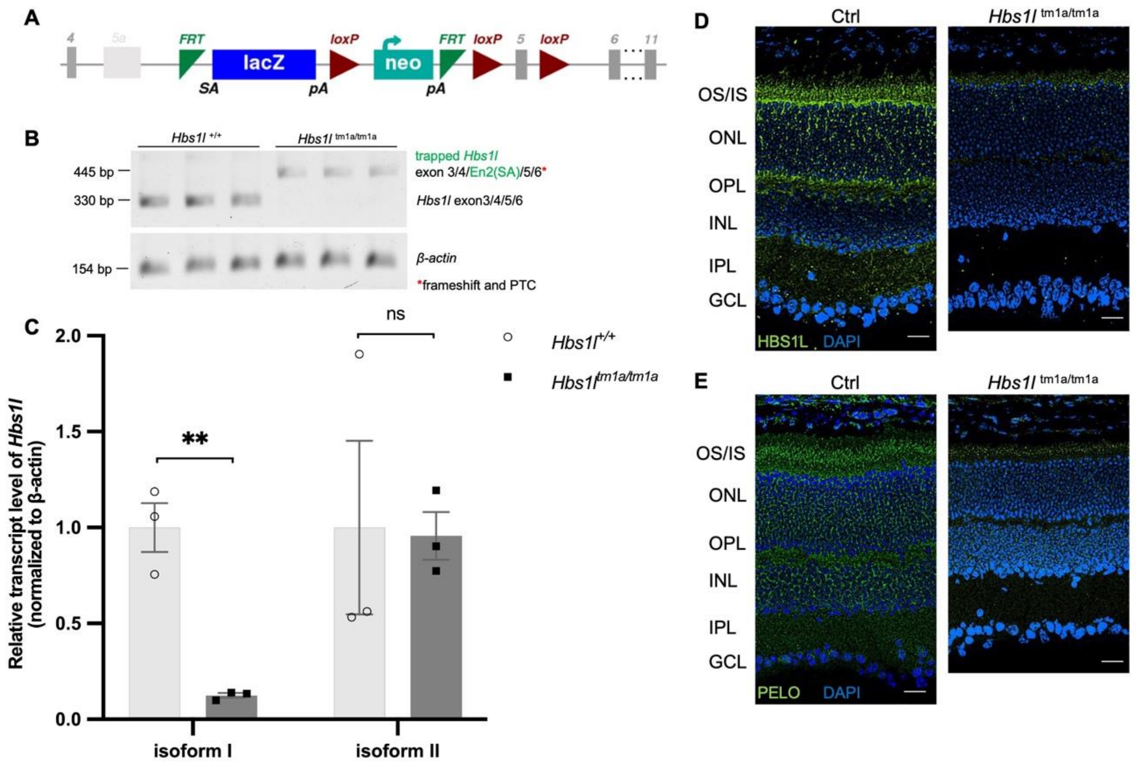

Fig. S4. Characterization of the *Hbs1l*$^{tm1a/tm1a}$ KO-first (hypomorph) mouse model at the transcript and protein level. (**A**) Schematic design of *Hbs1l* $^{tm1a/tm1a}$ KO-first (hypomorph) allele. (**B**) Splicing analysis of correctly spliced *Hbs1l* and trapped *Hbs1l* transcripts in eye tissues from 1-month-old control (Hbs1l$^{+/+}$) and *Hbs1l*$^{tm1a/tm1a}$ mice. β-actin was used as an input control. (**C**) Quantitative RT-PCR analysis of *Hbs1l* isoform I and isoform II using cDNA from eye tissues of 1-month-old control (*Hbs1l*$^{+/+}$) and *Hbs1l*$^{tm1a/tm1a}$ mice. Data were normalized to β-actin and the fold change in gene expression is relative to that of controls (*Hbs1l*$^{+/+}$) from each cross. Data represent mean ± S.E.M., *n*=3 per group. Two-tailed Student's t-test was used for statistical analysis. ***P* < 0.01. (**D, E**) Representative immunofluorescence microscopy images showing protein expression of Hbs1l (**D**) and Pelo (**E**) in the eye tissues of 1-month-old control (*Hbs1l*$^{+/+}$) and *Hbs1l*$^{tm1a/tm1a}$ mice. Please note that the retinal segments shown here are not sectioned at the level of optic nerve, thus location-wise, their retinal thickness might not be comparable because of their differential distance away from the optic nerve. Scale bar is 100 μm.

Disease Models & Mechanisms • DMM • Accepted manuscript

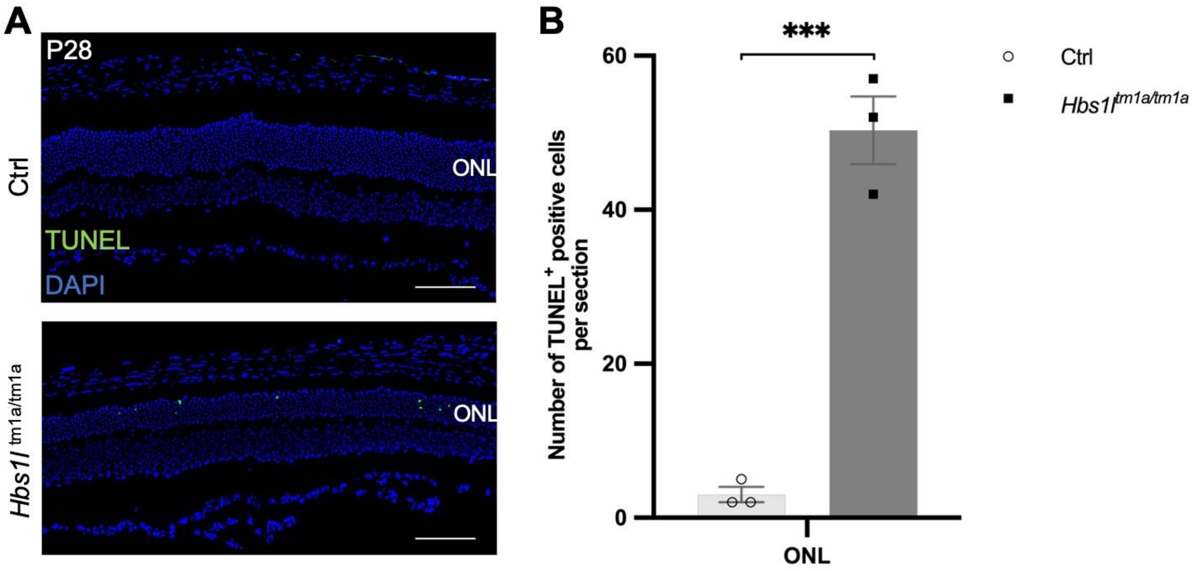

Fig. S5. Continued apoptosis of photoreceptor cells in *Hbs1l*$^{tm1a/tm1a}$ mice at 4 weeks of age. (A) Representative images of retinal sections from 4-week-old control (Hbs1l$^{+/+}$) and *Hbs1l*$^{tm1a/tm1a}$ mice. TUNEL-positive cells, green labelling; 4′,6-Diamidino-2-phenylindole counterstain for cell nuclei. Scale bar is 100 μm. **(B)** Histogram displaying the number of TUNEL-positive cells per section from 4-week-old control (Hbs1l$^{+/+}$) and *Hbs1l*$^{tm1a/tm1a}$ mice. Data represent mean ± S.E.M., n=3 per group. Two-tailed Student's t-test was used for statistical analysis. ***$P < 0.001$.

Disease Models & Mechanisms • DMM • Accepted manuscript

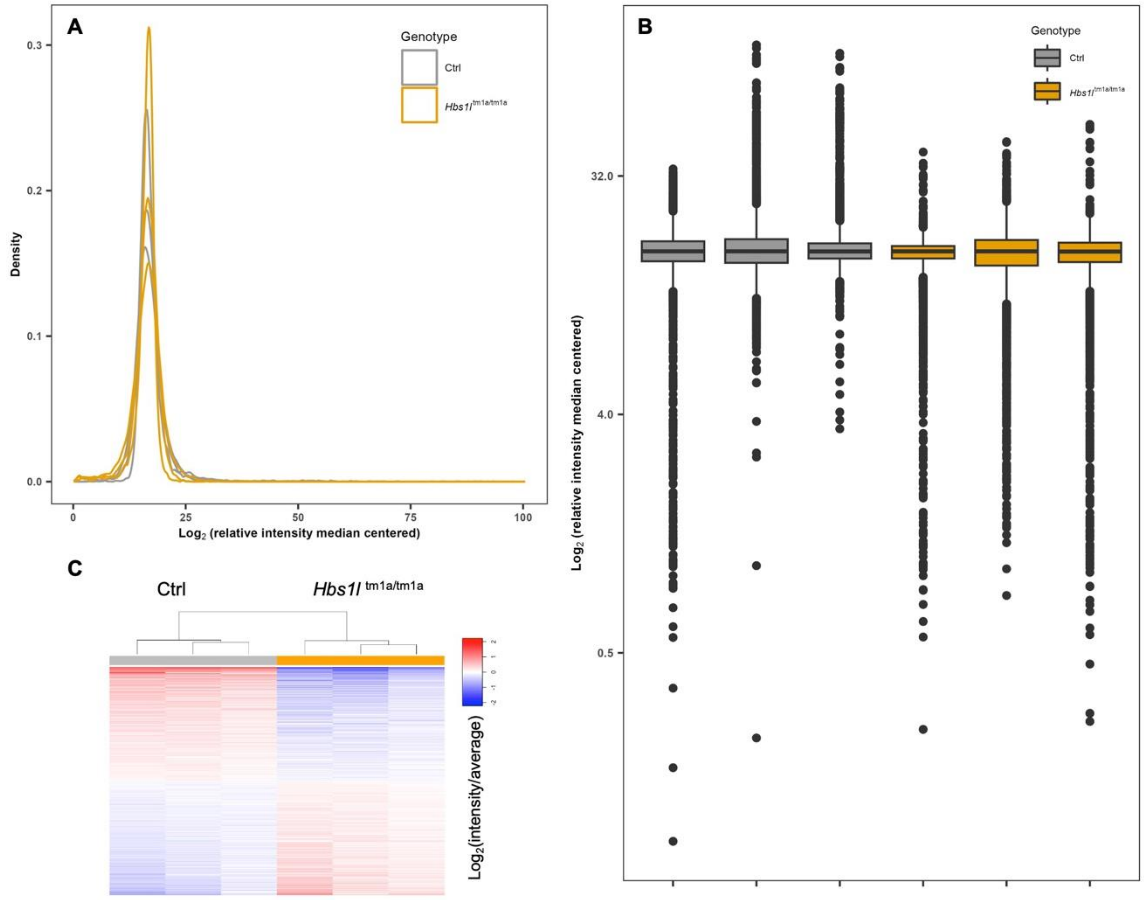

Fig. S6. Sample density, box plots of TMT experiments and heatmap analysis of proteins with differential abundance. A series of descriptive statistical analyses were performed to confirm the reproducibility of the data. The overall distribution of median-normalized and log transformed protein abundances were visualized in density (**A**) and box plots (**B**). The boxplots showed similar median and 95% confidence intervals. Moreover, comparison of density plots for each individual biological sample showed similar and highly overlapping patterns, with no major asymmetric bias, satisfying the normality assumption for further analysis. **(C)** heatmap of hierarchical clustering of the biological replicates (*n*=3 per group). Overall, these statistical metrics confirm the similarity in overall protein abundance distributions of individual biological replicates and demonstrate the reproducibility of measurements within sample groups.

Disease Models & Mechanisms • DMM • Accepted manuscript

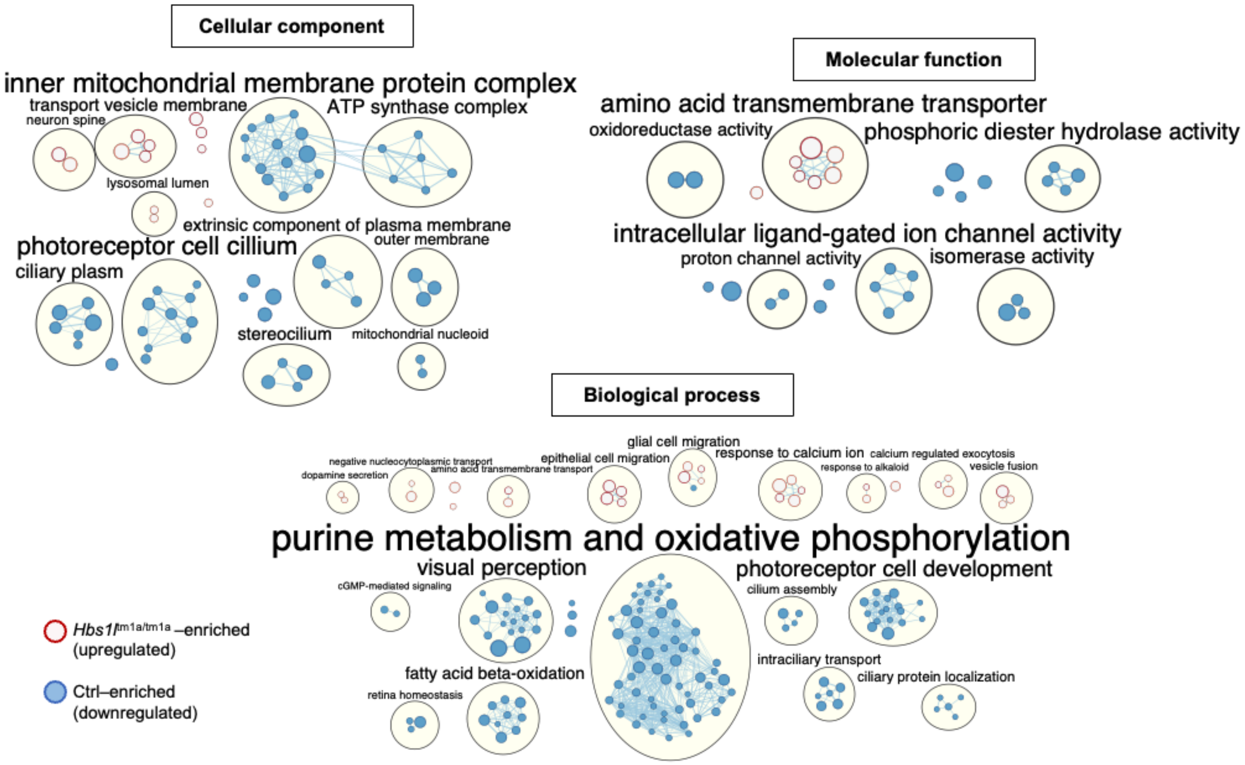

Fig. S7. Enrichment map of Gene Ontology (GO) cellular component, molecular function, and biological process clusters in *Hbs1l*$^{tm1a/tm1a}$ mice relative to littermate controls. This enrichment map was created with parameters FDR Q value < 0.01, and combined coefficient >0.375 with combined constant = 0.5. Red and blue nodes represent *Hbs1l*$^{tm1a/tm1a}$ and control-enriched GO terms, respectively. The size of a node indicates the size of each GO term, and the edge means two connected nodes share some genes. Nodes were manually laid out to form a clearer picture. Clusters of nodes were labeled using the AutoAnnotate Cytoscape application. Individual node labels were removed for clarity using the publication-ready button in EnrichmentMap.

Disease Models & Mechanisms • DMM • Accepted manuscript

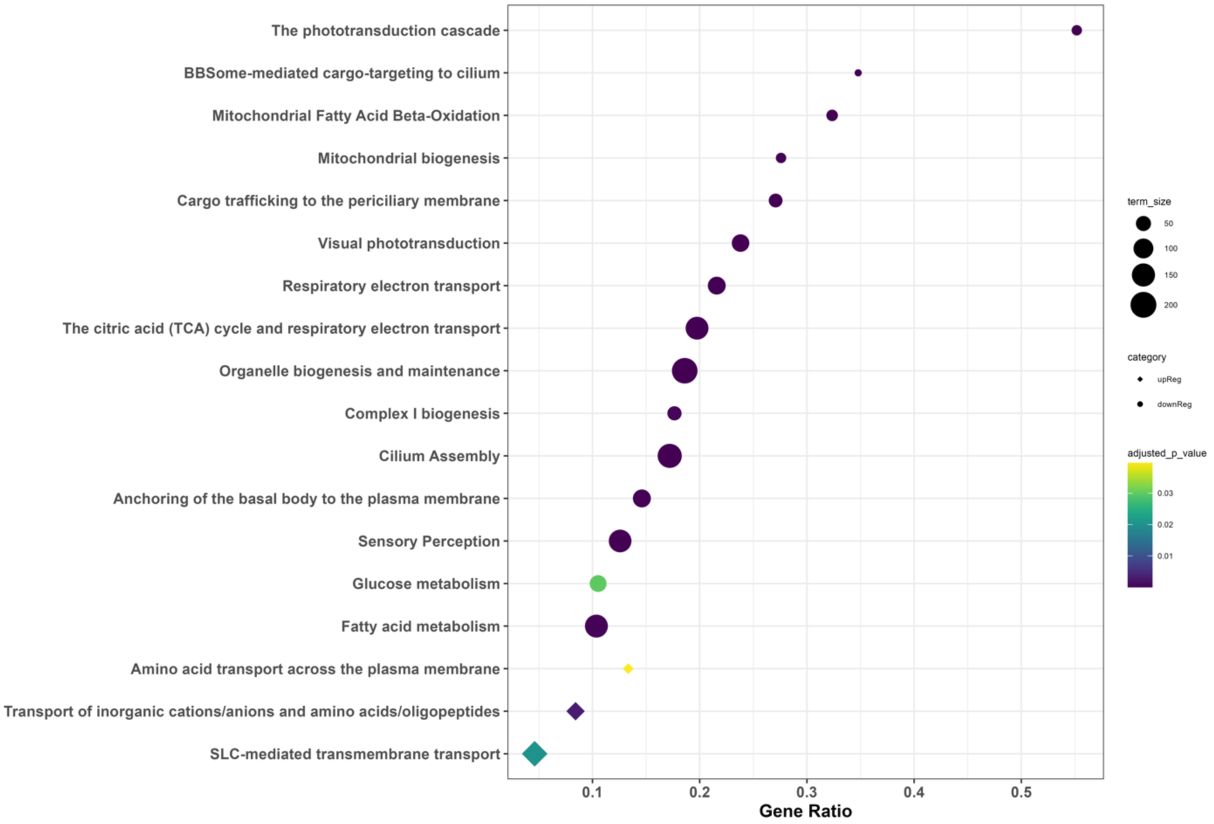

Fig. S8. Reactome pathway analysis of DMPs in *Hbs1l*$^{tm1a/tm1a}$ mice relative to littermate controls. A total of 645 DMPs (190 up regulated and 455 down regulated) were used for this analysis using g:Profiler (https://biit.cs.ut.ee/gprofiler). The top 15 most significantly enriched pathways from downregulated proteins and all three pathways from upregulated proteins were shown for illustration. The circles are colored by adjusted p value and the size of a circle indicates the gene size of each pathway.

Disease Models & Mechanisms • DMM • Accepted manuscript

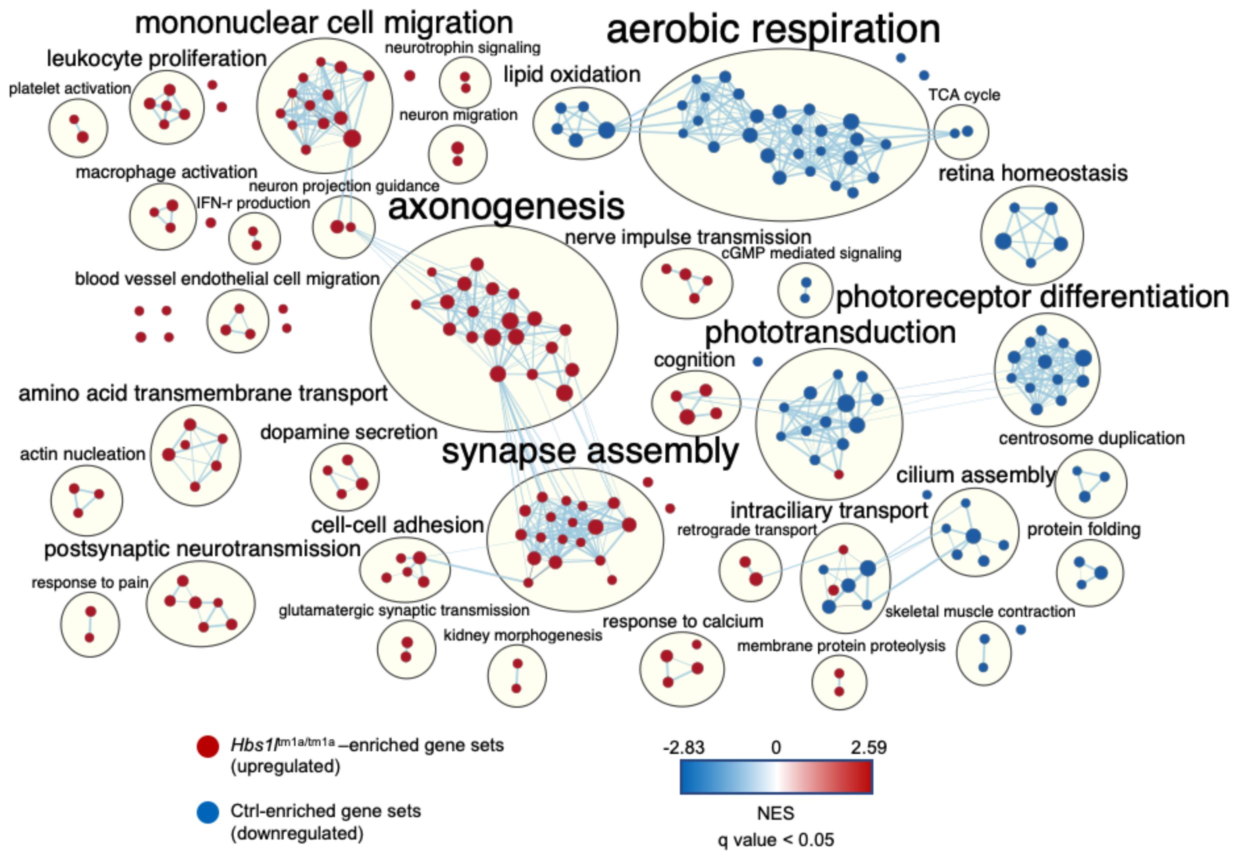

Fig. S9. Enrichment map of gene set enrichment analysis in *Hbs1l*$^{tm1a/tm1a}$ mice relative to littermate controls. This enrichment map was created with parameters FDR Q value < 0.05, and combined coefficient >0.5 with combined constant = 0.5. Nodes were colored using normalized enrichment score (NES). Red and blue nodes represent *Hbs1l*$^{tm1a/tm1a}$ and control-enriched gene sets, respectively. The size of a node indicates the size of each gene set, and the edge means two connected nodes share some genes. Nodes were manually laid out to form a clearer picture. Clusters of nodes were labeled using the AutoAnnotate Cytoscape application. Individual node labels were removed for clarity using the publication-ready button in EnrichmentMap.

Disease Models & Mechanisms • DMM • Accepted manuscript

Table S1. List of 8114 identified protein groups in retina samples of control and *Hbs1l*$^{tm1a/tm1a}$ mice with less than FDR 1% of proteins.

Available for download at
https://journals.biologists.com/dmm/article-lookup/doi/10.1242/dmm.050557#supplementary-data

Table S2. List of 649 differentially modulated proteins in *Hbs1l*$^{tm1a/tm1a}$ mice compared to controls. Proteins that met the p-value cut-off (≤0.05) and at least 50% difference were considered as differentially modulated proteins (DMPs).

Available for download at
https://journals.biologists.com/dmm/article-lookup/doi/10.1242/dmm.050557#supplementary-data

Table S3. Gene ontology and pathway enrichment analysis of DMPs in *Hbs1l*-deficient retina using g:Profiler. (A) Cellular component, (B) Molecular function, (C) Biological process, (D) Reactome pathway are listed.

Available for download at
https://journals.biologists.com/dmm/article-lookup/doi/10.1242/dmm.050557#supplementary-data

Disease Models & Mechanisms • DMM • Accepted manuscript
